# I lack ‘me-time’: The experiences of family caregivers of elders with Diabetes Mellitus in a selected village in South Africa

**DOI:** 10.4102/hsag.v27i0.2026

**Published:** 2022-11-10

**Authors:** Ipfi P. Mamatsharaga, Ntsieni S. Mashau, Jessica U. Damian

**Affiliations:** 1 Department of Public Health, Faculty of Health Sciences, University of Venda, Thohoyandou, South Africa

**Keywords:** coping strategies, Diabetes Mellitus, experiences, family caregivers, older people

## Abstract

**Background:**

Family caregiving is common in African countries where the family plays a crucial role in caring and supporting a family member in need of care. Most older people who suffer from chronic diseases, including Diabetes Mellitus, stay at home where family members and relatives are responsible for their day-to-day care.

**Aim:**

The purpose of this study was to explore the experiences of family caregivers of older people living with Diabetes Mellitus in a rural village in South Africa.

**Setting:**

This study was conducted in a village located in the Thulamela municipality of the Vhembe district, in the Limpopo province.

**Methods:**

A qualitative exploratory, descriptive research design was used. Purposive sampling was used, and data were collected using a semi-structured interview guide. Data saturation occurred after interviewing 17 participants and were analysed using thematic analysis. Trustworthiness was ensured through strategies of credibility, dependability, conformability, and transferability, and ethical principles were applied.

**Results:**

Three themes emerged: participants sacrificed time for work, time for self-care, and devised coping strategies. These findings revealed that family caregivers of older adults diagnosed with Diabetes Mellitus sacrificed their time in order to provide care. However, the findings further revealed that family caregivers devised strategies to cope through prayer and acceptance.

**Conclusion:**

The study revealed several challenges experienced by family caregivers of older people living with Diabetes Mellitus.

**Contribution:**

The National Department of Health, together with the civil society would gain an in-depth understanding of the burden of Diabetes Mellitus in the family setting and develop strategies to improve the situation.

## Introduction

According to Chisale (2018:3), family caregiving is an African way of life and constitutes Ubuntu. African communities live as one big family, providing care and support to one another in times of need or happiness. An African family includes all relatives who are related by birth or by marriage and extends to the neighbours. The characteristics of a family include involvement or contribution, unity, concern for each other, love and compassion towards each other. The family is always interconnected through the wisdom of the elderly people in that family. All these characteristics make it possible for an African family to provide care and support for each other. Although it is reiterated by Chisale (2018:5) that in Africa the caregiving role was previously provided by both males and females; however, elderly females in the family were responsible for providing day-to-day care to the sick. Family caregivers of the older people living with Diabetes Mellitus experience various challenges that may include the burden of monitoring sugar levels and assisting with activities of daily living (Baig, Benitez, Quinn 2015:4).

According to the World Health Organization (WHO 2016), Diabetes Mellitus is one non-communicable disease (NCD) that is a major source of morbidity and mortality worldwide. The number of adults living with Diabetes Mellitus globally in 2019 was estimated to be 463 million; this number is expected to rise to 578 million by the year 2030 (Health Systems Trust 2019:134). In South Africa, the prevalence of Diabetes Mellitus among the adult population of 15 years and older was estimated to increase from 5.5% in 2008 to 10.6% in 2017 (Health Systems Trust 2019:134). In the same report, it was indicated that the high prevalence of Diabetes Mellitus among adults is in the Western Cape, part of Northwest, and the metropolitan municipalities of KwaZulu-Natal (KZN), and the Eastern Cape. In the Limpopo province, the prevalence of Diabetes Mellitus in the Vhembe district is 13.2% (Health Systems Trust 2019:136).

According to Kalyani, Golden and Cefalu (2017:440), as the number of older people with Diabetes Mellitus increases, so does the complexity and difficulty in its management. These authors emphasised that as ageing is characterised by the degeneration of body cells, older people living with Diabetes Mellitus may suffer from cognitive dysfunction, immobility, and hearing and vision loss, which may affect self-care. Therefore, the provision of home-based care for older people living with Diabetes Mellitus becomes a mammoth task, especially for the family members.

Family caregivers play a fundamental role in the care of the terminally ill in many countries (International Diabetes Federation 2015). A study conducted in Ghana revealed that family caregiving in resource-limited settings is challenging and caregivers experience practical and emotional stress (Salifu, Almack & Caswell 2020:4). Being a caregiver has been associated with negative health impacts, psychological morbidity, social isolation, physical ill-health, and financial hardship.

In South Africa, most older people living with Diabetes Mellitus live with their family members at home because of the shortage of professional health workers and limited resources in public hospitals (National Department of Health 2001:2). In 2001, the South African Government through the Department of Health, developed a Home-based Care/Community-Based Care programme with the aim of involving the community members and non-governmental organisations (NGOs) through voluntary services, to provide care to people suffering from chronic diseases at home, in order to alleviate the already strained public health services (National Department of Health 2001:2). In 2011, the South African government through the Department of Health, further developed Ward-based Primary Healthcare Outreach Teams comprising healthcare professionals and community healthcare workers (CHWs) as a means of Primary Healthcare Re-engineering. Both programmes are responsible for providing healthcare services to communities and households (Department of Health 2014:12). This paper presents the findings of the study that explored the experiences of family caregivers of older people living with Diabetes Mellitus in a rural village of the Limpopo province in South Africa.

## Research methods and design

A qualitative exploratory descriptive research design was used. The study was conducted in a village located in a rural part of the Thulamela municipality of the Vhembe district, in the Limpopo province. The village comprises of 1 486 households, with 60.4% of the households headed by females and a 76.5% dependency ratio. The majority (97.1%) of the population speaks the Tshivenda language (Statistics South Africa 2022).

### Population and sampling

The target population for the study included all family caregivers of older people living with Diabetes Mellitus at the selected village, who were 18 years and above. A purposive sampling method was used to select the participants. Those who were responsible for the provision of home care to older persons diagnosed with Diabetes Mellitus, and were willing to participate in the study were included. Participants who were unable to provide consent and those who were not in the village were excluded. The sample size of 17 family caregivers of older people living with Diabetes Mellitus was determined by data saturation.

### Data-collection instrument

The development of a semi-structured open-ended interview guide for the face-to-face interviews was informed by Cohen and Crabtree’s (2006) qualitative research guideline. The interview guide contained questions on the participants’ socio-demographics, and a central question: ‘What are your experiences of caring for older people diagnosed with Diabetes Mellitus?’ This was followed by probing questions that were guided by the participant’s response (WHO 2014). The interview guide was developed in English and transcribed to Tshivenda by a language expert, after which it was used to conduct a pre-test with five participants who fitted the inclusion criteria. This process was to ensure that the aim of the study was not lost as a result of the transcription. It is important to note that the five participants selected for the pre-test formed part of the main study as they had given reliable information.

### Data-collection procedure

After obtaining permission to conduct the study from the local traditional leader of the selected village, a meeting was arranged with the manager of the Home-Based Care organisation (those who were responsible for the provision of care to older people living with Diabetes Mellitus in the village), to identify households with an older person living with Diabetes Mellitus. Eligible potential participants were identified by the volunteer Home-Based Caregivers (HBCs) during visitations along with the first author, they were introduced to the study for clarification, and to build a rapport between the researchers and the potential participants. They were assured that their information would remain confidential and that participation in the study was voluntary.

Potential family caregivers who were willing to participate in the study were given consent forms to sign. However, some of them gave verbal consent because they were unable to read and write. Appointments for interviews were made at a convenient time and place chosen by the participant. Socio-demographic information was obtained from the participants before data collection, and codes were used instead of names to ensure anonymity. Face-to-face in-depth individual interviews were conducted by the first author with the family caregivers. The first author had no day-to-day work contact with the participants, although she was a local community development practitioner by profession. However, prior meetings with the participants during arrangements for data collection played a major role in the building of relationships with the participants.

All interviews were conducted in the local language of Tshivenda by the first author who is very fluent in the language, which allowed participants to freely explain their experiences of providing home care to elderly persons who were diagnosed with Diabetes Mellitus. The interview discussion was audio-recorded with the permission of the participants, and participants were invited to inform the author to stop recording if they felt uncomfortable. Field notes were written on the observations made, and were written immediately after data collection to avoid distraction of the interview conversations between the participant and the first author. These field notes were included in the analysis, and they provided the context of the interview. The interviews lasted for about 45–60 min for each participant. Interviews were conducted over two months from November 2019 to December 2019.

### Data analysis

The audio-recorded interviews were transcribed verbatim to the English language by a trained transcriber. Creswell and Poth’s (2017) thematic analysis framework was used to analyse the data. The first author read the transcript line by line to capture all relevant points. Codes were derived from the data and a list was made. Similar codes were merged into themes and sub-themes. An independent coder who was experienced in qualitative research went through the themes and sub-themes to ensure that they retain the meaning of the experiences of caring for older people living with Diabetes Mellitus as expressed by participants. The independent coder analysed the transcription of the interviews and compared it with the subset of the researcher’s coding decision for coding consistency. A consensus discussion was held between the independent coder and the first author, and some themes were collapsed into each other.

### Measures to ensure trustworthiness

The following criteria were applied to ensure trustworthiness; credibility, dependability, transferability, and conformability (Polit & Beck 2016:182). The researcher was involved in a prolonged engagement with the participants to build trust and rapport. The researcher also ensured credibility through member-checking by presenting the findings to participants for them to verify if the information was a true reflection of what they shared during data collection. To ensure dependability, an independent coder examined the findings, interpretations, and recommendations, and attested that they reflected what the participants had said. A detailed description of the research study setting, methods, findings, and verbatim quotes from individual interviews were used in the study to ensure transferability.

### Ethical considerations

Ethical clearance (no. SHS/19/PH/24/1410) was obtained from the University of Venda, and permission to conduct the study was obtained from the traditional leader of the village. Written informed consent was obtained from the participants before the interview process. Participants were informed in their own language that the participation in the study was voluntary and that they were free to withdraw from the study at any time. No other person was allowed into the interview area during the process, and the participants were assured that only the authors will have access to the data. Audio records were kept in a safe locked cupboard and codes were used instead of participants’ real names to ensure confidentiality and anonymity.

## Results

The findings were informed by the participants and were divided into a socio-demographic profile, and themes and sub-themes.

### Socio-demographic profile

The study comprised 17 female family caregivers of older people living with Diabetes Mellitus. The age of the participants ranged from 26 to 60 years. All participants were Africans, and their home language was Tshivenda. Out of 17 participants, six were employed and one was a pensioner. The participants were related to the older person under their care; seven were children of the older person living with Diabetes Mellitus, six were daughters-in-law, three were wives, and one was the mother.

## Themes and sub-themes

Themes and sub-themes based on the challenges of family caregivers of older people living with Diabetes Mellitus are summarised in Table 1.

**TABLE 1:** Themes and sub-themes.

Themes	Sub-themes
Participants had no time for work	Financial problems Lack of resources
Participants sacrificed their time for selfcare	No time to engage in social activities Emotional stress
Coping strategies	Acceptance of the situation Prayers Support visits by home-based caregivers Support from relatives and friends

*Source:* Adapted from Mamatsharaga, I.P., 2020, ‘Challenges and coping strategies of caregivers caring for elderly people living with diabetes mellitus in a selected village of the Vhembe district, Limpopo province’. Dissertation, University of Venda, South Africa. Available from: http://hdl.handle.net/11602/1585

### Theme 1: Participants had no time for work

Participants explained that as a result of the caregiving, they could not engage in income-generating activities and therefore experienced financial constraints and lack of resources.

#### Sub-theme 1.1: Financial constraints

Participants reported that they experienced financial difficulties because they did not have the time to work or engage in income-generating activities. This hardship reflects in their inability to provide the basic needs that can help manage Diabetes Mellitus. Participants explained that some of the older people who they are providing care for do not receive an old age grant, and therefore there is no income to support the family. Although some participants explained that they try to participate in income-generating activities such as temporary jobs, they were still experiencing financial constraints to an extent that they would loan money from neighbours to buy food:

‘My mom is not receiving a grant, and it’s a challenge because she will say she wants fruit and vegetables and I have to buy. Where I am going to get the money because I am not working.’ (Participant 5, 29 years, employed)

‘… [*L*]ook now I have 3 children and also my mother-in-law, I don’t work, she has no grant so if I was working and I was going to hire a person of her age who will help me and also taking are of my children.’ (Participant 4, 38 years, unemployed)

‘I also do not have a permanent job and it is also a challenge because we do not afford to her healthy food every time and you find that there is no money at home she will have to make loan from neighbors so that we can buy food and repay back the money when I get paid and is not a lot.’ (Participant 14, 51 years, unemployed)

Participants in this study expressed the importance of receiving a stipend from the government for providing care to an older person at home; however, others preferred to be given healthy food parcels by the government:

‘We also need food parcels from the government because we don’t have enough money to buy a well-balanced diet for our elderly people living with Diabetes Mellitus.’ (Participant 3, 30 years, unemployed)

This study revealed that some extended families experience financial constraints because the wife who was a caregiver did not control the husband’s grant and moreover the grant was not enough to support the whole family:

‘My husband is receiving an old age grant, but I do not own the money because his brother’s wife is the one who is taking the money, and she is the one who is buying groceries here at home, and the grant is not enough to buy all the needs. I do not own my husband’s money, but I am the one who is taking care of him.’ (Participant 9, 32 years, unemployed)

#### Sub-theme 1.2: Lack of resources

The findings in this study further revealed that family caregivers of older people diagnosed with Diabetes Mellitus would often find themselves unable to pay for transport to carry an older person at night if their condition deteriorates and they become ill:

‘Yes! Sometimes we used to hire a car from those with cars, which is very expensive at night, like these diabetic attacks her mostly at night. I don’t work, and my grandmother is the only one getting a grant here at home; she has to buy food and also have money for transport to the hospital in case she will need to hire a car.’ (Participant 5, 29 years, employed)

‘The other thing is freedom of movement, I wish she could get the wheelchair that can help her to move in the house because her legs sometimes can lock, and she will be unable to move to the kitchen and also toilet, but if she has a wheelchair, I think it can help a lot.’ (Participant 13, 31 years, unemployed)

Participants further expressed that as they do not have time to work, they experience lack or inadequate supply of necessary medicines and relevant medical kits such as gloves that the caregivers should use when providing care to older persons diagnosed with Diabetes Mellitus at home:

‘My challenge is that if she goes to the clinic and does not get medication, we will have to buy the pills. Seriously, the government needs to do something. It is very much painful and disappointing.’ (Participant 2, 26 years, employed)

‘Hmm…the last one is that their medications and also gloves to use when you’re bathing them should be found in the clinic so that it can be simple for us to access them.’ (Participant 14, 51 years, unemployed)

### Theme 2: Participants sacrificed time for selfcare

Participants expressed that because of caring for older people at home, they do not find the time to care for themselves as they are overburdened with their caring activities. Caring for older people at home results in participants not having time for social activities and they end up suffering from emotional stress.

#### Sub-theme 2.1: No time to engage in social activities

Participants highlighted time-management constraints as another challenge they are facing. They were not being able to attend to their personal matters; as a result, they have no choice but to devote their time fully to taking care of their patients:

‘…. I try to balance everything, but I only lack self-time. I used to go to church, but time is no longer allowing me to attend church as I cannot go out and leave her alone because she needs attention. My shopping also is very hard as I only use weekends because there will be at least someone watching over her at home and even if I manage to go shopping, I don’t spend much time there as she needs me.’ (Participant 1, 39 years, employed)

#### Sub-theme 2.2: Emotional stress

The family caregivers indicated that they do not sleep well at night because some patients need to be taken care of at night and this included administering medication. This physical exhaustion has led to some of the participants experiencing emotional stress and developing high blood pressure:

‘You have to wake up and give them medication, sometimes they need cold water from the fridge to put their legs inside, and you end up not knowing what to do.’ (Participant 11, 31 years, employed)

Participants expressed mental exhaustion as the weight of their responsibility engulfs them:

‘People say I am mentally disturbed, and I am not, it is just that I have got a lot of things to attend to. I am also suffering from blood pressure due to the burden that I am having here at home and I have got no one to help me at least today we have got a visitor or a person that we can at least share our experiences with. I thought maybe I am the only one who is having this problem, you must come back again. Today I feel so much relieved; I am no longer that much stressed.’ (Participant 3, 30 years, unemployed)

Participants explained that they need counselling because they experience a lot of pressure which is emotionally exhausting. They further indicated the need for them to be trained in how to care for an elderly person diagnosed with Diabetes Mellitus.

‘… [*W*]e need to have a special counselor whom we can talk to because today I feel more relieved that I am talking to you because I don’t have anyone to share my secrets with.’ (Participant 13, 31 years)

‘… [*A*]t least they should give us counselling and teach us how to take care of them.’ (Participant 3, 30 years, unemployed)

### Theme 3: Coping strategies

Participants indicated that although the burden of caring feels too much for them, they found the courage to continue through acceptance of the caring role and prayer.

#### Sub-theme 3.1: Acceptance

The participants explained that they had come to accept the situation they found themselves in because it was the only way they could deal with the burden of caring for an older person.

‘… Sometimes tells you a lot of things and swearing at me but because I accept, I just keep quiet and then she will be fine and eat afterwards so is very disturbing if you fail to accept.’ (Participant 12, 32 years, unemployed)

#### Sub-theme 3.2: Prayers

In line with their religious faith, participants expressed that one of the major things that keeps them and even some of the patients going, is their faith in God through prayers.

‘With prayers, we pray a lot, it is normal to find people praying in the family to stabilize the situation and our hearts we pray a lot. Sometimes we even invite our pastor to come and pray with us so that will feel calm by the situation.’ (Participant 13, 31 years, unemployed)

#### Sub-theme 3.3: Support visits by home-based caregivers

Participants spoke in favour of the support visit from HBCs, which they described as crucial. They pointed out that the HBCs also motivate and help them to communicate with patients.

‘Home-based care is also helping a lot. They do come and visit their patient and sometimes they collect her medication from the clinic. The clinic provides us with medication even if sometimes there is a shortage of medication.’ (Participant 13, 31 years, unemployed)

#### Sub-theme 3.4: Support from relatives and friends

Occasional visits from friends and family were reported to help both the caregiver and the patient. If the visit is for the patient, the caregiver uses the opportunity to unwind.

‘Yes! Sometimes her sister and pastor do come and visit us. They give us counselling and console us as well as praying for us to cope with the situation.’ (Participant 9, 32 years, unemployed)

‘When a friend or a family member visit, it is a great feeling, we need them at almost everything, may be to talk to or just to see them.’ (Participant 3, 30 years, unemployed)

## Discussion

The present study explored the experiences of family caregivers of older people living with Diabetes Mellitus in a rural village in South Africa. The findings of this study revealed that participants had no time to work, and they sacrificed their time for selfcare to care for an older person diagnosed with Diabetes Mellitus. However, despite all the burden experienced because of the caregiving, participants were able to accept the situation through prayer and support from the HBCs, relatives, and friends.

### Participants had no time for work

Participants in this study expressed that they spend their time taking care of the older people in their households. The findings in this study revealed that participants do not have time to work and sacrificed their time for selfcare because they could not leave an older person living with Diabetes Mellitus alone at home.

A study conducted in the Limpopo province by Mphasha and Mothiba (2021:6), revealed that participants do not have time to attend to their personal needs because they devote their time entirely to care for the older people living with Diabetes Mellitus. Some of the older people living with Diabetes Mellitus need assistance with activities of daily living such as meal planning and proper preparation, bathing, and medical appointments (Mphasha & Mothiba 2021:6). According to Githaiga and Swatz (2017:6), family caregivers of people living with Diabetes Mellitus often feel abandoned as they have to carry the burden of caregiving alone and their entire time is taken up by the caregiving role. The report posits the kinship between time devoted to the care of an elder person living with Diabetes Mellitus and the quality of time dedicated to the general health and well-being of the caregiver (De Moor et al. 2017:6).

Financial constraint was a dominant challenge expressed by almost all the caregivers who participated in this study. It affects their ease of planning a healthy diet and transportation for ease of movement from one place to the other. This finding is supported by the global report on Diabetes by the World Health Organization (2016:1), which revealed the negative economic impact of caring for people living with Diabetes Mellitus on the family members.

Participants in this study highlighted income-related loss experienced because some had to sacrifice their jobs or could not get any chance to engage in income-generating activities because of time constraints. At the same time, some took up part-time jobs so that they could have time to be actively involved in the care of an older person diagnosed with Diabetes Mellitus at home. Inadequate financial support was found to be a limiting factor in the quality of care provided to older persons living with Diabetes Mellitus. Healthy food is one of the interventions to promote and support a healthy lifestyle for people living with Diabetes Mellitus (WHO 2016:50). Therefore, without any financial support from the government, it was difficult and frustrating for family caregivers of older people living with Diabetes Mellitus.

On the other hand, the findings of the current study revealed the unavailability of essential medical supplies such as gloves and other equipment required to assist an older person diagnosed with Diabetes Mellitus at home was a great challenge for the family caregivers. This finding is supported by Tamura, Omura, Toyoshima & Araki (2020:8), who indicated that without the medical supply of orthopaedic kits like wheelchairs and elbow crutches for those older persons who always encountered difficulties with their mobility it became more difficult for caregivers to help the older persons to move around at home (Kalyani et al. 2017:441). Lipka, Kończalska and Kędziora-Kornatowska (2020:328) go further to suggest the use of orthopaedic kits as a preventive measure against the development of diabetic foot in older people living with Diabetes Mellitus.

### Participants sacrificed time for selfcare

This study revealed that caregiving for an older person living with Diabetes Mellitus has a negative impact on the emotional health of participants. This study further revealed that participants reported ill-health like high blood pressure and depression, as a result of the stress they go through while carrying out their duties to help the older people living with Diabetes Mellitus. This finding corroborates with that of Salifu et al. (2020:4) regarding the effects of the challenges caregivers face in palliative care on their health.

The participants in this study expressed the need for professional counselling as a channel to let out their stress and anxieties. Family caregivers, as informal caregivers to older people living with Diabetes Mellitus need support from other family members in order not to feel isolated. Likewise, inadequate medical and social support, including training from health service officials, might hinder quality care for people living with Diabetes Mellitus because of their insufficient knowledge of health literacy surrounding diabetes care (Tu & Liao 2021:11; Salifu et al. 2020:8).

### Coping strategies

The findings of the current study revealed strategies adopted by the participants to cope with the identified challenges. The participants resorted to accepting the situation as their cross to bear and then pray to God and hope for a better daily outcome. Acceptance also helped them to ignore the verbal abuse they receive from some patients sometimes, which could result from the feeling of depression that the patient might be experiencing. Kalyani et al. (2017:441), described verbal abuse as one of the characteristics of geriatric syndrome highly symbolic with older people. In this study, participants expressed appreciation and relief when relatives and friends visited them and assisted in the caregiving role of older people with Diabetes Mellitus. They further indicated that the HBCs from a local Home-Based Organisation also visit them and take care of the older person living with Diabetes Mellitus. Although the HBCs do not come every day, participants expressed great relief at their visits.

### Limitations

The study was conducted in a rural village and focused on the experiences of family caregivers of older people diagnosed with Diabetes Mellitus in a rural village. Further research exploring the experiences of older people living with Diabetes Mellitus and receiving home care from family members would be of great value.

## Conclusion

This study revealed that family caregivers of older people living with Diabetes Mellitus do not have time to engage in income-generating activities and to care for themselves because of the caregiving role. However, the family caregivers in this study also expressed the factors that encourage them to cope with the burden of caregiving.

Recommendations as a result of the study include counselling of family caregivers by professionals, such as social workers and psychologists. Caring for older people living with Diabetes Mellitus should be a collaborative effort between the family, primary healthcare centres, and the civil society and the National Department of Health.

There is a clear need for urgent interventions from the government and NGOs as there is a possibility that more caregivers may be experiencing the same challenges. The National Department of Health, Social Development and the Civil Society could use the findings of the study to develop strategies and measures to alleviate the burden of home care of older persons living with Diabetes Mellitus by family members.
